# Lung Nodules in Melanoma Patients: Morphologic Criteria to Differentiate Non-Metastatic and Metastatic Lesions

**DOI:** 10.3390/diagnostics11050837

**Published:** 2021-05-07

**Authors:** Simone Alexandra Stadelmann, Christian Blüthgen, Gianluca Milanese, Thi Dan Linh Nguyen-Kim, Julia-Tatjana Maul, Reinhard Dummer, Thomas Frauenfelder, Matthias Eberhard

**Affiliations:** 1 Institute of Diagnostic and Interventional Radiology, University Hospital Zurich, Raemistrasse 100, 8091 Zurich, Switzerland; Simone.Stadelmann@gmx.net (S.A.S.); Christian.Bluethgen@usz.ch (C.B.); ThiDanLinh.Nguyen-Kim@usz.ch (T.D.L.N.-K.); thomas.frauenfelder@usz.ch (T.F.); 2 Department of Medicine and Surgery (DiMeC), University of Parma, 43126 Parma, Italy; gianluca.milanese@unipr.it; 3 Department of Dermatology, University Hospital Zurich, Raemistrasse 100, 8091 Zurich, Switzerland; Julia-Tatjana.Maul@usz.ch (J.-T.M.); Reinhard.Dummer@usz.ch (R.D.)

**Keywords:** pulmonary nodules, malignant melanoma, multi-detector computed tomography

## Abstract

Lung nodules are frequent findings in chest computed tomography (CT) in patients with metastatic melanoma. In this study, we assessed the frequency and compared morphologic differences of metastases and benign nodules. We retrospectively evaluated 85 patients with melanoma (AJCC stage III or IV). Inclusion criteria were ≤20 lung nodules and follow-up using CT ≥183 days after baseline. Lung nodules were evaluated for size and morphology. Nodules with significant growth, nodule regression in line with RECIST assessment or histologic confirmation were judged to be metastases. A total of 438 lung nodules were evaluated, of which 68% were metastases. At least one metastasis was found in 78% of patients. A 10 mm diameter cut-off (used for RECIST) showed a specificity of 95% and a sensitivity of 20% for diagnosing metastases. Central location (*n* = 122) was more common in metastatic nodules (*p* = 0.009). Subsolid morphology (*n* = 53) was more frequent (*p* < 0.001), and calcifications (*n* = 13) were solely found in non-metastatic lung nodules (*p* < 0.001). Our data show that lung nodules are prevalent in about two-thirds of melanoma patients (AJCC stage III/IV) and the majority are metastases. Even though we found a few morphologic indicators for metastatic or non-metastatic lung nodules, morphology has limited value to predict the presence of lung metastases.

## 1. Introduction

Melanoma is a common skin cancer with an incidence rate of 19.7 per 100,000 and a death rate of 2.7 per 100,000 [1]. Melanoma has a high potential of metastasizing lymphatogenously or hematogenously to any organ [2]. In particular, the lungs are frequently affected, as their involvement has been reported in 10–40% of patients [3,4]. Pulmonary metastatic involvement is associated with short median overall survival (range 6–13 months), thus reflecting the aggressiveness of such advanced oncologic disease [5,6]. Overall survival can be increased by pulmonary metastasectomy, although this procedure can only be performed in selected cases [4]. Routine cross-sectional imaging with computed tomography (CT), positron emission tomography-CT (PET/CT) or magnetic resonance tomography for disease staging is not recommended in asymptomatic patients with American Joint Committee on Cancer (AJCC) stage 0–II due to a high number of false positive findings [7]. In contrast, imaging (such as chest CT) is recommended for staging and surveillance of disease in patients with melanoma stage IIb or higher [7,8].

Lung nodules are frequent findings on chest CT scans [9,10,11]; however, most of them are benign [12,13]. In the specific setting of staging chest CT in melanoma patients, differentiating between metastatic and non-metastatic nodules at baseline CT scans is difficult although of high clinical and prognostic relevance.

Using Response Evaluation Criteria in Solid Tumors (RECIST) 1.1 or immune RECIST (iRECIST) to assess tumor response to therapy, a 10 mm long axis diameter cut-off is used to define target lesions and, therefore, also applicable in lung nodules [8,14]. However, currently it is unknown how specific this threshold is when applied in lung nodules. Choosing a non-metastatic lung nodule with a diameter of at least 10 mm for RECIST assessment at baseline could lead to a wrong classification of treatment response in patient follow-up.

This study aims (I) to describe the frequency of lung nodules and lung metastases in a cohort of patients with AJCC stage III or IV, (II) to examine morphologic and size differences between metastatic and non-metastatic lung nodules and (III) to assess the sensitivity and specificity for a 10 mm long axis diameter cut-off in pulmonary nodules used to define target lesions according to the RECIST criteria.

## 2. Methods

### 2.1. Overview and Study Design

This retrospective study was approved by the local Ethics Committee and conducted according to the principles of the Declaration of Helsinki. Patient consent was waived. We retrospectively evaluated a cohort of 253 advanced stage melanoma patients (AJCC stage III or IV) undergoing baseline chest CT scans before treatment initiation at the University Hospital Zurich between 01/2010 and 12/2017. These chest CT scans were screened for the presence of lung nodules by a radiologist with four years of experience in thoracic radiology (initials omitted for the review) applying Computer Aided Detection (CAD) (Syngo.via, Siemens Healthineers, Forchheim, Germany) as a second reader.

### 2.2. Patients—Baseline Characteristics and Follow-Up

Sex, age, date of baseline and follow-up CT scans, melanoma stage (as defined by AJCC and TNM Classification of Malignant Tumors), primary tumor site, smoking status and additional diagnoses at the time of diagnosis were retrospectively recorded. Melanoma disease progression according to the RECIST classification noted in the electronic medical record was recorded at the time of the last follow-up CT scan.

### 2.3. Baseline CT Scan Protocol

Baseline contrast-enhanced chest CT scans were either performed using a second-generation dual-source 128 slice scanner (SOMATOM Definition Flash, Siemens Healthineers, Forchheim, Germany), a second-generation single-source 128 slice scanner (SOMATOM Definition Edge, Siemens Healthineers, Forchheim, Germany) or a third-generation dual-source 192 slice scanner (SOMATOM Force, Siemens Healthineers, Forchheim, Germany) at a tube voltage of 100 kVp or 120 kVp. Tube current time product was adjusted to patient habitus using automated attenuation-based tube current modulation. The rotation time was 0.28 s or 0.25 s. The following reconstruction parameters were used: sharp tissue convolution kernel, slice increment of 1.5 mm and slice thickness of 2.0 mm.

### 2.4. Lung Nodules

**Figure 1:**
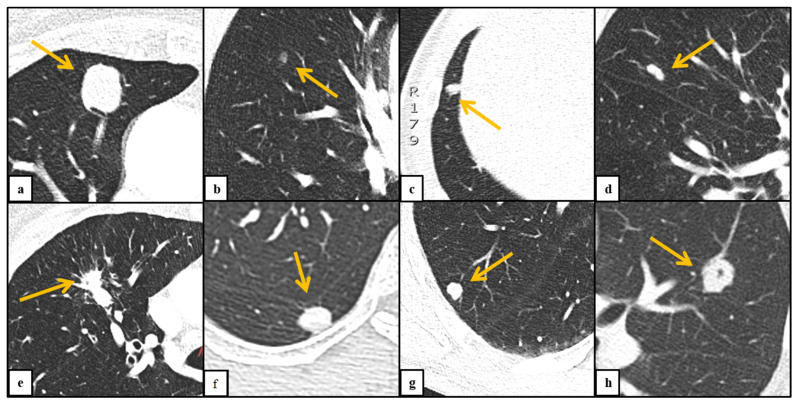
Characteristics of lung nodules. (**a**): solid nodule; (**b**): part-solid nodule; (**c**): ill-defined margin; (**d**): lobulated margin; (**e**): spiculated margin; (**f**): pleural retraction; (**g**): feeding vessel; (**h**): air bronchogram.

Baseline lung nodules were assessed for size, morphology and location by one radiologist (C.B., four years of experience in thoracic radiology; Figure 1). Long axis, perpendicular short axis and average diameter were measured on axial images. Semi-automated segmentation (Syngo.via, Siemens Healthineers, Forchheim, Germany) allowed volumetric measurements of lung nodules. Sphericity of lung nodules was calculated by the ratio of short axis diameter to the long axis diameter. Nodules were classified as solid, part-solid or non-solid as previously described by the Fleischner Society [15]. According to the nodules’ contour, margins were classified as ill-defined (margin irregular and poor), well-defined (linear demarcation), spiculated (margin radiating) and lobulated (margin undulated). Pleural retraction, evidence of air bronchogram, vessel attachment to nodules and nodule calcification were recorded. Multiple nodules organized as a group within the same lobe were defined as grouped and single nodules were defined as solitary. Peripheral location was defined as the outer 1/3 of the lung.

### 2.5. Lung Nodules on Follow-Up Chest CT Scan

According to the methodology used by Lee et al. [16], patients were considered as having pulmonary metastases if any baseline nodules were either pathologically confirmed as metastases, had increased in size at subsequent follow-up CT or had decreased after chemotherapy in accordance with RECIST evaluation. If nodules decreased in size while the RECIST classification of this patient was stable or progressive disease, nodules were categorized as benign nodules. A diameter change of ≥2 mm in either the long or short axis was considered as a significant size increase or decrease according to the Fleischner recommendations [17].

### 2.6. Statistics

Normality of continuous data was assessed using the Shapiro–Wilk test. Non-normal distributed continuous data are shown as median and inter-quartile range (IQR). Fisher’s exact test, Pearson’s chi-squared test and Mann–Whitney U test were used where appropriate. Receiver Operator Characteristic (ROC) and Area Under the Curve (AUC) were used to predict metastases based on lung nodule sizes. The AUC is shown with a 95% confidence interval (95% CI). ROC curves were compared using DeLong’s test. Statistics were computed using IBM SPSS, version 25. A two-tailed *p* < 0.05 was considered as statistically significant.

## 3. Results

### 3.1. Patients

**Figure 2:**
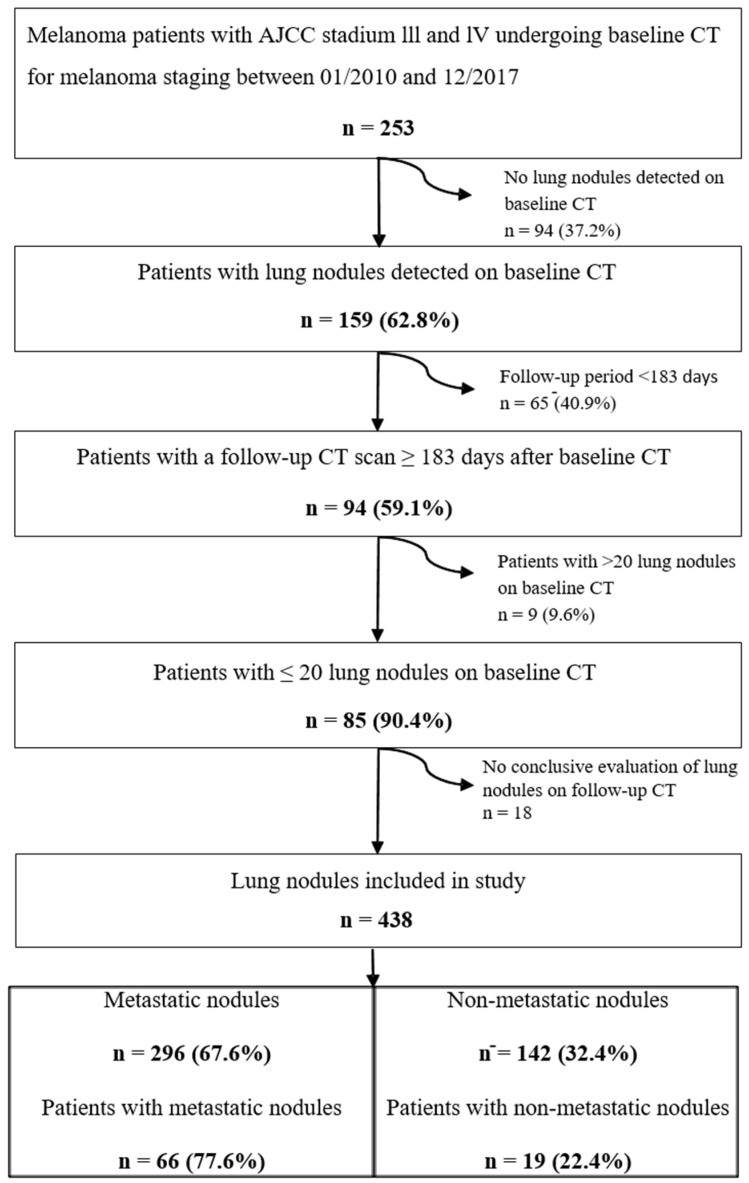
Flow chart illustrating derivation of our final study cohort. American Joint Committee on Cancer (AJCC). Computed Tomography (CT).

Lung nodules were detected on CT scans of 159 patients (63%). Eighty-five patients (median age, 58 years, 32 females) had follow-up CT conducted ≥183 days after baseline CT (median follow-up period, 672 days, range 186–3012) and a maximum number of 20 lung nodules per patient and, thus, were included for further assessment in this study (Figure 2). Seventy-one percent of patients were never-smokers (*n* = 60). Further baseline characteristics are shown in Table 1.

### 3.2. Evaluation of Lung Nodules

A total of 438 lung nodules were included (Figure 2). The median number of lung nodules per patient was 4 (interquartile range 2–7). Lung nodules characteristics are reported in Table 2. Half of the lung nodules were found in the lower lobes (*n* = 218; 50%) and just over half were detected in the right lung (*n* = 253; 58%). The median long axis diameter was 5.5 mm (IQR: 4.2–7.8 mm), median average diameter was 4.7 (IQR: 3.6–6.7 mm) and median volume was 53 mm^3^ (range 24–144 mm^3^).

### 3.3. Metastatic Lung Nodules

Sixty-eight percent of lung nodules (*n* = 296) were metastases. Most lung metastases resolved during follow-up (*n* = 210; 71%), 15% decreased in size (*n* = 44), 14% increased in size (*n* = 40) and 1% were resected (*n* = 2). At least one metastasis was found in the majority of patients (*n* = 66, 78%). The median number of metastases per patient was 2 (interquartile range 1–5). Our results indicate that 94% of patients with ≥3 lung nodules (*n* = 53) had at least one metastasis, compared to 50% of patients with ≤2 lung nodules (*n* = 32). Most metastases were found in the periphery of the lung (68%).

### 3.4. Indicators for Metastatic or Non-Metastatic Nodules

**Figure 3:**
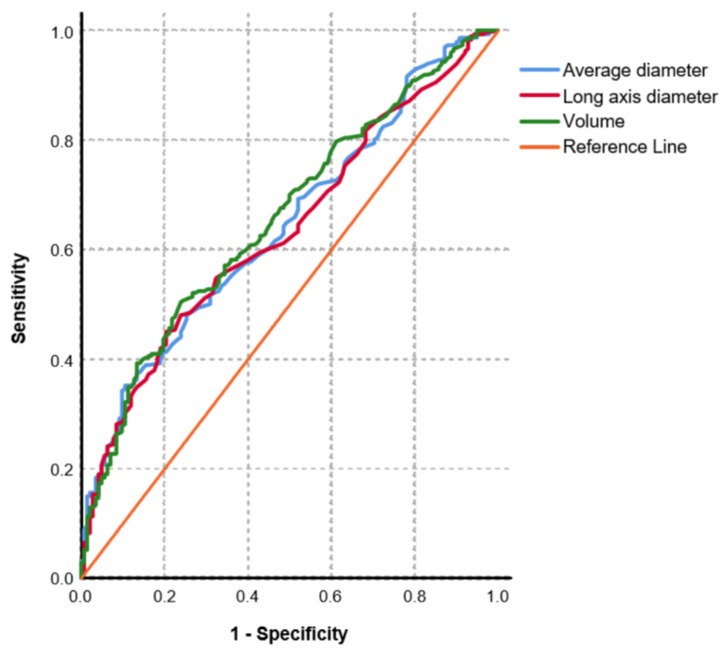
Receiver Operating Characteristic (ROC) curve illustrating the Area Under the Curve (AUC) for average nodule diameter (blue), maximum long axis diameter (red) and nodule volume (green) to predict metastases. There were no significant differences between ROC curves calculated with DeLong’s test (*p* = 0.059–0.325).

Metastatic nodules were slightly larger compared to non-metastatic nodules (median long axis diameter: 6.0, IQR: 4.4–8.8 mm versus 5.2, IQR: 3.7–6.1 mm; median average diameter 5.1, IQR: 3.7–7.4 versus 4.2, IQR: 3.3–5.3 and median volume: 71 mm^3^, IQR: 28–202 mm^3^ versus 36, IQR: 19–69 mm^3^; *p* < 0.001 for both) on baseline CT scans. Furthermore, metastases were slightly rounder with a slightly higher median sphericity index (0.8, IQR: 0.6–0.8 versus 0.7, IQR: 0.6–0.8; *p* = 0.038). Figure 3 shows ROC curves for long axis diameter, average diameter and volume measurements of lung nodules to predict metastases. Long axis diameter measurements showed an AUC of 0.64 (95% CI: 0.58–0.69). Using a long axis diameter cut-off value of 5 mm, specificity and sensitivity to diagnose metastases were low with 48% and 63%, respectively. Using a cut-off value of 10 mm for long axis diameter (as proposed in the RECIST criteria), specificity and sensitivity to detect a metastasis were 95% and 20%, respectively. The AUCs to predict metastases were slightly higher for average diameter (AUC: 0.64, 95% CI: 0.59–0.70) and volume measurements (AUC: 0.66, 95% CI: 0.61–0.71), however, without statistical significance (*p* = 0.325 and *p* = 0.059, respectively). Centrally located nodules were more likely to represent metastases (77% of central nodules versus 64% of peripheral nodules; *p* = 0.009). Subsolid nodules were more frequently non-metastatic (*p* < 0.001). All calcified nodules (*n* = 13) were non-metastatic (*p* < 0.001). Margins of lung nodules, presence of grouped nodules, pleural retraction, air bronchogram and the feeding vessel sign did not show significant differences between metastatic and non-metastatic nodules.

## 4. Discussion

Even though the presence of lung metastases is likely in advanced stage melanoma patients [7,18], the high number of non-metastatic lung nodules frequently detected on CT scans suggests that a relevant proportion of lung nodules detected on CT scans of melanoma patients are non-metastatic [19]. Our data indicate that (I) 63% of melanoma patients (AJCC stage III or IV) undergoing staging chest CT showed lung nodules and 78% of those patients presented with at least one lung metastasis; (II) 68% of all lung nodules were metastases, with central location and larger size increasing the likelihood for the presence of metastases. In contrast, subsolid nodules were more frequently non-metastatic and all calcified nodules were non-metastatic; (III) a cut-off value of 10 mm used to define a target lesion using the RECIST criteria yielded a high specificity of 95%.

The estimated risk of pulmonary metastases for melanoma patients (AJCC I–IV) is given as 13% at 5 years, derived from a large prospective comprehensive cancer center database of 14,057 melanoma patients [20]. In patients with non-pulmonary cancer, Hanamiya et al. demonstrated that 75% of patients had lung nodules, and 80% of these were benign [21]. However, less than 10 patients of the overall cohort of 308 patients had melanoma, as one study group including patients with melanoma, sarcoma, or testicular carcinoma comprised 10 patients [21]. In our study (melanoma patients with AJCC stage III or IV), we found a slightly lower frequency of lung nodules detected on staging CT (63%), with about 2/3 of nodules representing metastasis (68%).

Previously, it was shown that lung nodule size (>5 mm) and multiplicity of lung nodules correlate with the presence of metastases [22]. Our results indicate that lung nodules with increased size are more likely to be metastatic (median volume 71 mm^3^ compared to 36 mm^3^; median long axis diameter 6.0 mm compared to 5.2 mm). However, size differences between metastatic and non-metastatic lung nodules were very small and size may be an insufficient predictor of metastasis. Accordingly, in our study, a long axis diameter cut-off value of 5 mm showed a low accuracy to diagnose lung metastasis (specificity, 48%; sensitivity, 63%). More interestingly, we found a long axis diameter ≥10 mm to have a high specificity of 95% (sensitivity, 20%), indicating that the cut-off value used to define a target lesion using the RECIST criteria correctly identifies a lung metastasis in most patients. On the one hand, this result is reassuring as the choice of a non-metastatic nodule as a target lesion would hinder correct tumor response assessment during follow-up. On the other hand, the low sensitivity of this cut-off shows its limited value for the differentiation of metastatic and non-metastatic lung nodules. Furthermore, our study showed that the multiplicity of lung nodules can also be used as an indicator for the presence of lung metastases. Our results indicate that 94% of patients with ≥3 lung nodules had at least one metastasis, compared to only 50% of patients with ≤2 lung nodules. In line with previous reports, we found that most metastases are more frequently located in the periphery of the lung (68%) [23]. However, central lung nodules had a slightly higher likelihood to be metastases (77% versus 64%). Association of calcification of any type (diffuse, central, laminated, popcorn patterns) with benign dignity of lung nodules is widely reported and support our findings that all calcified nodules were non-metastatic lung nodules [9]. We found subsolid nodules were more likely to be non-metastatic. Complementing our results, melanoma metastases have been reported to rarely present with subsolid morphology [23,24,25]. However, subsolid nodules carry a higher risk for pulmonary adenocarcinoma [25]. Overall, our study shows that 22% of patients with lung nodules at chest CT performed for screening of melanoma patients at AJCC stage III or IV do not have lung metastases. Correct identification of non-metastatic nodules would have important implications for patient treatment. However, our results show that morphologic criteria have a limited value for the differentiation of metastatic and non-metastatic lung nodules. Therefore, biopsy and/or follow-up imaging with chest CT or implementation of PET/CT [26,27] have to be considered when indeterminate lung nodules are found. Further research should investigate whether patients with indeterminate lung nodules may benefit from an increased frequency of follow-up chest CT. Additionally, our results show the need for further application and research of artificial intelligence-based algorithms [28,29]. Convolutional neuronal networks may have the potential to overcome the limitations of sheer visual assessment of lung nodule morphology. These neuronal networks are able to analyze feature maps in which the density of each pixel/voxel of an image is weighted by convolution matrices and different matrices can be applied for specific tasks, such as blurring, sharpening or edge detection [30]. These algorithms enable the assessment of texture metrics, which are not visually perceptible. Previously, Li et al. reported that three different deep learning architectures reached a lung nodule classification accuracy between 68 and 99.6% and a detection accuracy between 81 and 94% [29].

Our study has some limitations. Firstly, being a single center study, the number of patients eligible for inclusion in the study, as well as demographic distribution among patients, was restricted and may be susceptible for referral bias. Secondly, only advanced clinical stages AJCC III or IV melanoma patients were included. These stages require the presence of regional lymph node or lymphatic metastases and/or distant non-pulmonary metastases [7]. With these conditions given, the probability of pulmonary metastases development is increased [7]. Thirdly, lung nodules with significant size changes over the follow-up period might not only be of metastatic origin but also from infections or primary lung cancer, among others. To minimize the erroneous judgement of infectious nodules as metastases, lung nodules that decreased in size or disappeared during follow-up were only judged metastases when size decrease was in accordance with the overall RECIST evaluation [16]. Fourthly, application of our results may be reduced in regions with endemic infectious diseases resulting in multiple lung nodules such as tuberculosis or histoplasmosis.

## 5. Conclusions

Our data show that lung nodules are prevalent in about two-thirds of patients with advanced stage melanoma (AJCC III or IV) and that the majority (about 70%) of these are metastases. Even though we found a few morphologic indicators for metastatic or non-metastatic lung nodules (size, multiplicity of nodules, central location, density and calcification), morphology has a limited value to predict the presence of lung metastases.

## Figures and Tables

**Table 1:** Baseline characteristics of patients.

Baseline Characteristics of Patients (*n* = 85)		
**Sex**		
Male	53	62%
Female	32	38%
**Age (years)**	58	32–80
**Smoking status**		
Never-smokers	60	71%
Current smoker	11	13%
Former smokers	14	17%
**Melanoma staging**		
T1	8	9%
T2	13	15%
T3	27	32%
T4	19	22%
n/a	18	21%
N0	9	11%
N1	20	24%
N2	22	26%
N3	21	25%
n/a	13	15%
M0	22	26%
M1	63	74%
**Primary tumor site**		
Cutaneous	71	84%
Head and Neck	15	18%
Body trunk	26	31%
Upper extremities	12	14%
Lower extremities	18	21%
Connective tissue (choroidal)	2	2%
Non-keratinized stratified squamous epithelium (vaginal)	2	2%
Unknown	10	12%

Abbreviation: AJCC, American Joint Committee on Cancer staging system. Categorical data are given as count (percentage) and continuous data are given as median (range).

**Table 2:** Characteristics of lung nodules. Morphology of lung nodules and differences between metastatic and non-metastatic nodules.

	All Lung Nodules		Non-Metastatic Lesions		Metastases		*p*-Value
	*N* = 438		*N* = 142	32%	*N* = 296	68%	
**Side**							
Right	253	58%	82	58%	171	58%	1.00
Left	185	42%	60	42%	125	42%	
**Lobe**							
Lower lobe	218	50%	65	46%	153	52%	0.410
Upper lobe	177	40%	64	45%	113	38%	
Middle lobe	43	10%	13	9%	30	10%	
**Location**							
Central	122	28%	28	20%	94	32%	0.009
Periphery	316	72%	114	80%	202	68%	
**Morphology**							
Subsolid	53	12%	29	20%	24	8%	<0.001
Non-solid	8	2%	7	5%	1	0.3%	
Part-solid	45	10%	22	15%	23	8%	
Solid	385	88%	113	80%	272	92%	
**Margin**							
Ill-defined	24	6%	7	6%	17	6%	0.821
Well-defined	387	88%	129	91%	258	87%	
Lobulated	22	5%	5	4%	17	26%	
Spiculated	5	1%	1	1%	4	1%	
**Grouping**							
Solitary	425	97%	138	97%	287	97%	1.000
Grouped	13	3%	4	3%	9	3%	
**Pleura retraction**							
No	431	98%	142	100%	289	98%	0.102
Yes	7	2%	0	0%	7	2%	
**Air bronchogram**							
No	436	99.5%	142	100%	294	99%	1.000
Yes	2	0.5%	0	0%	2	1%	
**Feeding Vessel**							
No	214	49%	76	54%	138	47%	0.186
Yes	224	51%	66	46%	158	53%	
**Calcification**							
No	425	97.0%	129	91%	296	100%	<0.001
Yes	13	3.0%	13	9%	0	0%	

## Data Availability

Data available on request due to privacy and ethical restrictions.
